# Risk factors for central hypoventilation in anti-*N*-methyl-D-aspartate receptor encephalitis

**DOI:** 10.3389/fimmu.2025.1718192

**Published:** 2025-12-11

**Authors:** Xuan Zou, Xi-yue Jing, Guan-en Zhou

**Affiliations:** 1 Department of Neurology, Tianjin Huanhu Hospital, Tianjin University, Tianjin, China; 2 Clinical College of Neurology, Neurosurgery and Neurorehabilitation, Tianjin Medical University, Tianjin, China; 3 Institute of Neurosurgery, Tianjin Huanhu Hospital, Tianjin University, Tianjin, China

**Keywords:** anti- *N*-methyl-D-aspartate receptor encephalitis, central hypoventilation, risk factors, consciousness disorders, cerebrospinal fluid antibody titer

## Abstract

**Objective:**

This study aims to identify the risk factors for central hypoventilation in patients with anti-*N*-methyl-D-aspartate receptor (anti-NMDAR) encephalitis.

**Methods:**

Patients with anti-NMDAR encephalitis who were hospitalized between January 2020 and January 2025 were identified from the Tianjin Huanhu Hospital database, affiliated with Tianjin University. Patients were categorized into a central hypoventilation group and a non-central hypoventilation group, and potential risk factors were collected. Statistical methods were used to compare the two groups and identify risk factors.

**Results:**

The analysis revealed that the central hypoventilation group exhibited a higher incidence of involuntary movements (*χ²*=5.662, *P*=0.017), lower admission Glasgow Coma Scale (GCS) scores (*Z*=-3.749, *P*<0.001), and higher cerebrospinal fluid (CSF) antibody titers compared with the non-hypoventilation group (*Z*=-3.344, *P*=0.001). Multivariate logistic regression analysis identified only admission GCS score and CSF antibody titer as independent risk factors. A lower GCS score [*P*=0.018; *OR* (*95% CI*): 0.556 (0.343, 0.903)] and a higher CSF antibody titer [*P*=0.048; *OR* (*95% CI*): <0.001 (0.000, 0.903)] significantly increased the likelihood of central hypoventilation.

**Conclusion:**

Disturbances in consciousness and elevated CSF antibody titers are risk factors for central hypoventilation in patients with anti-NMDAR encephalitis. It is imperative to closely monitor the respiratory status and oxygenation levels of patients identified as high-risk, conduct regular blood gas analyses, and implement respiratory support and more aggressive immunotherapy when necessary.

## 1 Introduction

Anti-*N*-methyl-D-aspartate (NMDAR) encephalitis is the most prevalent form of autoimmune encephalitis. It is characterized by the presence of anti-NMDAR antibodies that bind to extracellular conformational epitopes in the NR1/NR2 heteromers of the NMDAR. These antibodies are often detected in the CSF or serum of young female patients with ovarian teratoma (1). In contrast, male patients are less commonly affected and rarely present with concurrent tumors. In addition to tumors, herpes simplex encephalitis is a known trigger of NMDAR autoimmunity (2).

The clinical symptoms of anti-NMDAR encephalitis include cognitive impairment, epilepsy, mental or behavioral abnormalities, and involuntary movements. Some patients may exhibit characteristic autonomic dysfunction, such as arrhythmia, increased salivation, hypotension, or central fever (3). Among these complications, central hypoventilation is the most severe. Patients with central hypoventilation often require airway management and mechanical ventilation. Without timely intervention, the prognosis is poor (4–7).

Although recovery is slow (2), most patients with severe anti-NMDAR encephalitis ultimately achieve good long-term outcomes after early, active, and sustained immunotherapy and life support (8). Therefore, identifying risk factors for central hypoventilation is crucial in clinical practice. Early identification of high-risk patients enables closer monitoring, early detection of hypoventilation, and the timely initiation of respiratory support and aggressive immunotherapy to improve outcomes.

## 2 Materials and methods

### 2.1 Inclusion criteria and exclusion criteria

Patients with anti-NMDAR encephalitis hospitalized between January 2020 and January 2025 were identified from the Tianjin Huanhu Hospital database, affiliated with Tianjin University. Patients were categorized into a central hypoventilation group and a non-central hypoventilation group.

Inclusion criteria: (i) Antibodies in the CSF were detected by cell-based assay (CBA) and tissue-based assay (TBA). All enrolled patients tested positive for anti-NMDAR antibodies and met the diagnostic criteria for anti-NMDAR encephalitis (9); 2. The criterion for central hypoventilation met at least one of the following conditions: (1) partial pressure of oxygen (PO_2_)<60 mmHg in supine and quiet position without supplemental O_2_; (2) partial pressure of carbon dioxide (PCO_2_) > 55 mmHg for ≥10 minutes during sleep or PaCO2 ≥ 45 mmHg during wakefulness (10), which improved only with high-flow oxygen therapy, airway maintenance, or ventilator-assisted breathing therapy.

Exclusion criteria: (i) Patients with negative CSF antibody results or missing records; (ii) Patients with hypoventilation caused by other conditions, such as severe pneumonia or cardiac insufficiency.

### 2.2 Collect clinical data of patients

Data on potential risk factors for central hypoventilation were collected, including age, gender, history of prodromal infection, tumor status, clinical symptoms (mental or behavioral abnormalities, epilepsy, cognitive impairment, involuntary movements), degree of consciousness disorder (admission GCS score), CSF antibody titer, CSF leukocyte count, CSF immunoglobulin G (CSF-IgG), presence of magnetic resonance imaging (MRI) lesions, and timing of immunotherapy.

### 2.3 Statistical analysis methods

For variables that follow a normal distribution, this study presents the mean ± standard deviation ( $\bar{x}\pm s$) to describe their centralized trend and discrete trend, and employs a t-test to analyze the differences between the two groups. For variables that do not conform to a normal distribution, the median and quartile range [*Median (Quartile Range)*] are used to describe their centralized trend and discrete trend, with a nonparametric test to assess the differences between the two groups. For categorical variables, this study uses the number of cases (constituent ratio) [n (%)] to describe, and uses chi-square test to analyze the differences between the two groups. Multivariate logistic regression [lo*git*(*π*)=*β*_0_+*β*_1_*X*_1_+ ⋯ +*β_P_X_P_*] was used to identify factors associated with central hypoventilation (11). Odds ratios (ORs) and 95% confidence intervals (*95%* CIs) were calculated to assess the influence of independent variables on central hypoventilation. The Receiver Operating Characteristic (ROC) curve was generated using the selected risk factors, and the area under the curve (AUC) was calculated to evaluate the model’s accuracy (12). A *P* value less than 0.05 was considered statistically significant.

## 3 Results

### 3.1 Included samples

A total of 42 patients with anti-NMDAR encephalitis were enrolled in this study. Among them, the central hypoventilation group comprised 9 patients (21.4%), including one patient who required high-flow oxygen inhalation, two patients who required airway opening, and six patients who required both airway opening and ventilator-assisted breathing. Central hypoventilation occurred mostly during the catatonic and hyperkinetic phase. The non-central hypoventilation group included 33 patients (78.6%).

### 3.2 Comparison of clinical data between the two groups

#### 3.2.1 Basic information

The median age of patients in the central hypoventilation group was 33.00 (21.00, 40.00) years, with 2 male patients (22.2%) and 7 female patients (77.8%). In this group, 4 patients (44.4%) had a history of prodromal infection, and 3 patients (33.3%) had tumors. In the non-central hypoventilation group, the median age was 38.00 (25.50, 55.00) years, consisting of 17 male patients (51.5%) and 16 female patients (48.5%). In this group, 15 patients (45.5%) had a history of prodromal infection, and 3 patients (9.1%) had tumors. There were no significant differences between the two groups in age (*Z*=-1.227*, P*=0.224), gender (*χ2 =* 1.410*, P*=0.235), prodromal infection history (*χ2 =* 0.000, *P*=1.000), or tumor status (*χ2 =* 1.703, *P*=0.192).

#### 3.2.2 Clinical symptoms

In the central hypoventilation group, 8 patients (88.9%) exhibited mental or behavioral abnormalities, 7 (77.8%) had epilepsy, 3 (33.3%) had cognitive impairment, and 4 (44.4%) had involuntary movements. In the non-central hypoventilation group, 16 patients (48.5%) had mental or behavioral abnormalities, 13 (39.4%) had epilepsy, 11 (33.3%) had cognitive impairment, and 2 (6.1%) had involuntary movements. There were no significant differences between the two groups in mental or behavioral abnormalities (*χ2 =* 3.208, *P*=0.073), epilepsy (*χ2 =* 2.780, *P*=0.095), or cognitive impairment (*χ2 =* 0.000, *P*=1.000). However, involuntary movements were significantly more common in the central hypoventilation group (*χ2 =* 5.662, *P=*0.017).

#### 3.2.3 Degree of consciousness disorder [admission (GCS) scores]

The median GCS score in the central hypoventilation group was 9.00 (8.00, 10.00), compared with 15.00 (13.00, 15.00) in the non-central hypoventilation group. Patients in the central hypoventilation group had significantly more severe disturbances of consciousness (*Z*=-3.749*, P*<0.001).

#### 3.2.4 Laboratory indicators

In the central hypoventilation group, the median CSF antibody titer was 1:32 (1:10, 1:100), the CSF leukocyte count was ( 41.33±50.95)×10^6^/L, and the CSF-IgG level was ( 65.95±73.81) mg/L. In the non-central hypoventilation group, the median CSF antibody titer was 1:3.2 (1:1, 1:10), the CSF leukocyte count was of ( 49.76±60.29)×10^6^/L, and the CSF-IgG level was ( 74.46±64.58) mg/L. The CSF antibody titer was significantly higher in the central hypoventilation group (*Z*=-3.344*, P*=0.001). No significant differences were found in CSF leukocyte count (*t*=-0.383, *P*=0.704) or CSF-IgG level (*t*=-0.340, *P*=0.736).

#### 3.2.5 Presence of MRI lesions

In the central hypoventilation group, 5 patients (55.6%) exhibited MRI lesions, while 4 patients (44.4%) did not. In the non-central hypoventilation group, 22 patients (66.7%) had lesions and 11 patients (33.3%) did not. There was no significant difference between the two groups (*χ2 =* 0.050, *P=*0.823).

#### 3.2.6 Timing of immunotherapy

The time from onset to immunotherapy initiation was 9.00 (3.00, 26.00) days in the central hypoventilation group and 14.00 (7.50, 31.50) days in the non-central hypoventilation group. The difference was not statistically significant (Z=-1.229, P=0.224).

Please see **Table 1** for detailed comparisons.

**Table 1:** Comparison of clinical data between the two groups.

Variable	Central hypoventilation group (n=9)	Non-central hypoventilation group (n=33)	Statistics	*P*
Age [year, median (quartile range)]	33.00 (21.00, 40.00)	38.00 (25.50, 55.00)	-1.227	0.224 ^a^
Male patients [n (%)]	2 (22.2)	17 (51.5)	1.410	0.235^b^
Prodromal infection [n (%)]	4 (44.4)	15 (45.5)	0.000	1.000 ^b^
Combined with tumor [n (%)]	3 (33.3)	3 (9.1)	1.703	0.192 ^b^
Clinical symptoms [n (%)]				
mental or behavioral abnormalities	8 (88.9)	16 (48.5)	3.208	0.073^b^
epilepsy	7 (77.8)	13 (39.4)	2.780	0.095^b^
cognitive impairment	3 (33.3)	11 (33.3)	0.000	1.000 ^b^
involuntary movements	4 (44.4)	2 (6.1)	5.662	0.017 ^b^
GCS score at admission [median (quartile range)]	9.00 (8.00, 10.00)	15.00 (13.00, 15.00)	-3.749	<0.001 ^a^
Laboratory indicators				
CSF antibody titer [median (quartile range)]	1:32 (1:10, 1:100)	1: 3.2 (1:1, 1:10)	-3.344	0.001^a^
CSF leucocyte count [×10^6^/L, ( $\bar{x}\pm s$)]	41.33±50.95	49.76±60.29	*-*0.383	0.704 ^c^
CSF-IgG [mg/L, ( $\bar{x}\pm s$)]	65.95±73.81	74.46±64.58	-0.340	0.736 ^c^
Presence of MRI lesions [n (%)]	5 (55.6)	22 (66.7)	0.050	0.823 ^b^
Timing of immunotherapy (day)	9.00 (3.00, 26.00)	14.00 (7.50, 31.50)	-1.299	0.224 ^a^

Statistics: a: nonparametric test; b: chi-square test; c: t-test. GCS, Glasgow Coma Scale; CSF, cerebrospinal fluid; IgG, glycoprotein G; MRI, Magnetic Resonance Imaging.

### 3.2 Multivariate logistic regression analysis

Variables identified as statistically significant in the univariate analysis (involuntary movements, admission GCS score, and CSF antibody titer) were included in the multivariate logistic regression. The results showed that both admission GCS score and CSF antibody titer were independent risk factors for central hypoventilation. A lower GCS score was associated with a higher likelihood of central hypoventilation [*P=0.018*; *OR (95% CI)*: 0.556 (0.343, 0.903)]. A higher CSF antibody titer was also associated with a greater likelihood of central hypoventilation [*P*=0.048; *OR (95% CI)*: <0.001 (0.000, 0.903)]. Please refer to **Table 2** for the multivariate logistic regression results.

**Table 2:** Multivariate logistic regression analysis.

Variable	*β*	*SE*	Wald χ2	*P*	*OR (95% CI)*
involuntary movements	3.406	1.886	3.260	0.071	30.153 (0.747, 1216.396)
GCS score at admission	-0.586	0.247	5.637	0.018	0.556 (0.343, 0.903)
CSF antibody titer	-14.749	7.473	3.895	0.048	<0.001 (0.000, 0.903)

GCS, Glasgow Coma Scale; CSF, cerebrospinal fluid.

### 3.3 Calculate AUC

**Figure 1:**
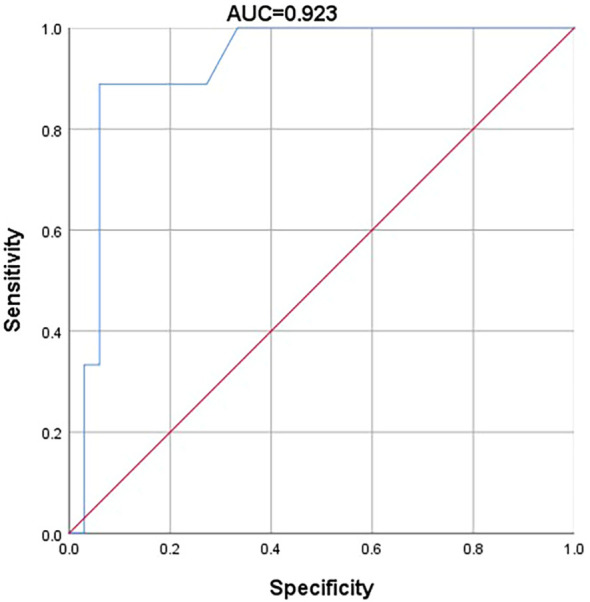
Multivariate logistic regression ROC curves of risk factors for central hypoventilation in patients with anti-NMDAR encephalitis.

The ROC curve was generated using the logistic regression model based on the independent risk factors (**Figure 1**). The AUC for the admission GCS score and CSF antibody titer was 0.923 (*95% CI*=0.834–1.000, *P*<0.001), indicating high predictive accuracy.

### 3.4 Conclusions

Compared with the non-central hypoventilation group, the central hypoventilation group showed a higher prevalence of involuntary movements (*χ2 =* 5.662, *P=*0.017), more severe disturbances of consciousness (*Z*=-3.749*, P*<0.001), and higher CSF antibody titers (*Z*=-3.344*, P*=0.001). However, only the degree of consciousness disturbance and CSF antibody titer were identified as independent risk factors for central hypoventilation. Specifically, a lower GCS score [p=0.018; *OR (95% CI)*: 0.556 (0.343, 0.903)] and a higher CSF antibody titer [p=0.048; *OR (95% CI)*: <0.001 (0.000, 0.903)] were associated with increased risk.

## 4 Discussion

Most patients with anti-NMDAR encephalitis experience five clinical stages: the prodromal phase, psychotic and/or seizure phase, unresponsive and/or catatonic phase, hyperkinetic phase, and gradual recovery phase (13). Central hypoventilation is a distinctive manifestation of anti-NMDAR encephalitis and typically occurs during the tension and hyperactivity phases (13). It usually appears within the first 2 months of the disease course (14–16), but may persist for longer durations. For example, one case report described a middle-aged female patient with anti-NMDAR encephalitis who developed persistent idiopathic central sleep apnea for 7 months. Her respiratory status improved significantly after treatment with intravenous methylprednisolone (IVGC) and intravenous immunoglobulin (IVIg), followed by rituximab induction therapy (16).

Reports from the Perelman School of Medicine at the University of Pennsylvania indicate that 40%–60% of adult patients with anti-NMDAR encephalitis experience central hypoventilation, ^(14-16) a^ proportion considerably higher than that reported in Chinese cohorts. In a study by Xu et al., only 19.4% of patients developed central hypoventilation (17). In our study, 21.4% of patients exhibited central hypoventilation, which is consistent with previous domestic findings. Beyond possible racial differences, the absence of a standardized definition of central hypoventilation in anti-NMDAR encephalitis may also contribute to the variability across studies.

Central hypoventilation is closely associated with severe autonomic dysfunction (3, 16, 18). This association is important because autonomic dysfunction suggests simultaneous involvement of respiratory autonomic pathways. He et al. conducted a study including 161 patients with autoimmune encephalitis, 85 of whom had anti-NMDAR encephalitis, and found that symptoms of autonomic dysfunction—such as sinus arrhythmia, pollakiuria/uroclepsia, fever, constipation, urinary retention, hyperhidrosis, hypersalivation, hypotension, and early satiety or emesis—were linked to a higher incidence of central hypoventilation (3).

The NMDAR plays a key role in multiple CNS functions, including the regulation of respiratory centers (19). Some studies suggest that central hypoventilation in anti-NMDAR encephalitis may be explained by the high density of NMDA-NR1 receptors in the Kölliker–Fuse nuclei (KFn), a brainstem region essential for respiratory modulation (15, 20). All modulation appears to depend on the integrity of the glutamatergic neurons within the Kölliker–Fuse (KF) region (21). However, we propose that central hypoventilation may not result solely from KF injury. There may also be more extensive involvement of other brainstem respiratory centers or nuclei, such as the pre-Bötzinger complex (preBötC) and the retrotrapezoid nucleus/parafacial respiratory group (RTN/pFRG) (22).

The literature on risk factors for central hypoventilation is limited. One study reported that older age at onset was associated with a higher likelihood of central hypoventilation (6). However, our study did not identify age as a contributing factor. Instead, we found that disturbances of consciousness and CSF antibody titer were independent risk factors.

Previous studies have shown that central hypoventilation frequently occurs in patients with anti-NMDAR encephalitis who exhibit disturbances of consciousness (9, 23). Such patients often experience severe neurological dysfunction (7, 14) and poorer outcomes (4, 6). However, the literature has not clearly established whether disturbances of consciousness directly constitute a risk factor for central hypoventilation. Our results suggest that they do indeed serve as an independent risk factor.

The KF region not only mediates inspiratory–expiratory phase transitions and gates eupneic motor discharges in the vagal nerves, but it also plays a crucial role in maintaining arousal and consciousness (24). Therefore, we speculate that disturbances of consciousness in anti-NMDAR encephalitis may be associated with injury to the KF region. Such injury may lead simultaneously to disturbances of consciousness and central hypoventilation. Furthermore, disturbance of consciousness may indicate damage to the extensive ascending reticular activating system (ARAS) within the brainstem, suggesting that other respiratory centers in the reticular structure may also be affected.

CSF antibody titer was also closely associated with central hypoventilation. Wang et al. studied 108 patients with anti-NMDAR encephalitis and found that the prevalence of central hypoventilation was higher in the high-titer group compared with the low-titer group (p=0.006) (25). In another study of 192 patients, Zhang et al. reported that those with teratoma had higher anti-NMDAR antibody titers than non-teratoma patients, and the incidence of central hypoventilation (52.4% vs. 17%, *P*<0.001) and decreased consciousness (71.4% vs. 31.3%, *P*=0.002) was significantly higher (26). Although these studies support a correlation between CSF antibody titer and central hypoventilation, they did not designate antibody titer as an independent risk factor. In our study, CSF antibody titer—along with disturbance of consciousness—was identified as an independent risk factor.

Elevated CSF antibody titers correlate with disease severity, including the degree of consciousness disturbance. Patients with high antibody titers often exhibit more severe clinical symptoms (7, 27, 28) and are more likely to develop disturbances of consciousness (29). As CSF antibody titers increase, the likelihood of damage to surface antigens of brainstem respiratory neurons may also increase. Greater injury to these respiratory centers may predispose patients to central hypoventilation.

## 5 Conclusion

Our findings indicate that more severe disturbances of consciousness [*P*=0.018; *OR (95% CI)*: 0.556 (0.343, 0.903)] and higher CSF antibody titers [p=0.048; *OR (95% CI)*: <0.001 (0.000, 0.903)] are associated with an increased likelihood of central hypoventilation in patients with anti-NMDAR encephalitis. The AUC for the admission GCS score and CSF antibody titer was 0.923 (95% CI=0.834–1.000, *P*<0.001), indicating a high level of accuracy in the predictive model.

Although previous studies have noted correlations between disturbances of consciousness, CSF antibody titer, and central hypoventilation, none have identified these variables as independent risk factors (23, 25, 26). Our study not only establishes both as independent risk factors for central hypoventilation in patients with anti-NMDAR encephalitis but also delves into the potential connections and underlying mechanisms between these factors.

Given the poor prognosis associated with central hypoventilation in anti-NMDAR encephalitis, neurologists should closely monitor the respiratory status and oxygenation of high-risk patients. Regular blood gas analyses and timely respiratory support are essential. More aggressive immunotherapy may be necessary, including early intensive first-line immunotherapy (7, 30, 31), and prompt transition to second-line treatment when the response to first-line therapy is inadequate (3).

## 6 Deficiency and inspiration

Anti-NMDAR encephalitis is a relatively rare autoimmune disease of the CNS, and only a subset of patients develop central hypoventilation. Consequently, the number of cases in this study is relatively small, which may influence the statistical findings. Future research should focus on the continued accumulation of cases. Moreover, this study is a single-center retrospective analysis, utilizing data obtained from the one of the largest neurology hospitals in China over the past 6 years. As such, it may better represent the characteristics of the patient population in North China.

## Data Availability

The raw data supporting the conclusions of this article will be made available by the authors, without undue reservation.
